# Integrated multispectral imaging, germination phenotype, and transcriptomic analysis provide insights into seed vigor responsive mechanisms in quinoa under artificial accelerated aging

**DOI:** 10.3389/fpls.2024.1435154

**Published:** 2024-09-30

**Authors:** Huifang Yan, Zhao Zhang, Yanzhen Lv, Yuting Nie

**Affiliations:** ^1^ College of Grassland Science, Qingdao Agricultural University, Qingdao, China; ^2^ Key Laboratory of National Forestry and Grassland Administration on Grassland Resources and Ecology in the Yellow River Delta, Qingdao, China

**Keywords:** quinoa, seed vigor, transcriptome, flavonoid biosynthesis, carbon metabolism, energy supply

## Abstract

Seed vigor is an important trait closely related to improved seed quality and long-term germplasm conservation, and it gradually decreases during storage, which has become a major concern for agriculture. However, the underlying regulatory mechanisms of seed vigor loss in terms of genes remain largely unknown in quinoa. Here, two cultivars of quinoa seeds with different storage performance, Longli No.4 (L4) and Longli No.1 (L1), were subjected to transcriptome sequencing to decipher the pathways and genes possibly related to vigor loss under artificial aging. Multispectral imaging features and germination phenotypes showed significantly less seed vigor loss in L1 than in L4, indicating L1 seeds having stronger aging resistance and storability. Totally, 272 and 75 differentially expressed genes (DEGs) were, respectively, identified in L4 and L1 during aging. Transcriptomic analysis further revealed the differences in metabolic pathways, especially, flavonoid biosynthesis, TCA cycle, and terpenoid backbone biosynthesis were significantly enriched in L4 seeds, while carbon metabolism in L1 seeds, which involved key genes such as *CHS*, *CHI*, *AACT*, *ENO1*, *IDH*, *NADP-ME*, and *HAO2L*. It indicated that the adverse effects on flavonoids and terpenoids induced by aging might be the significant reasons for more vigor loss in storage sensitive seeds, whereas storage tolerant seeds had a stronger ability to maintain carbon metabolism and energy supply. These findings elucidated the underlying molecular mechanism of seed vigor loss in quinoa, which also provided novel insights into improving seed vigor through modern molecular breeding strategies.

## 1 Introduction

Quinoa (*Chenopodium quinoa* Willd.) is a promising crop to improve food security, which is considered as a complementary species to traditional crops in the future agriculture, such as soybean [*Glycine max* (L.) Merr.], wheat (*Triticum aestivum* L.), maize (*Zea mays* L.), and rice (*Oryza sativa* L.) (Cui et al., 2023). Quinoa also has very important and extensive industrial application values. Its starch can be used in pharmaceutical industry and food processing industry due to the good gel, water absorption, emulsification and stability (Ren et al., 2023). Meanwhile, its flavonoids and saponin extracts also have great potential in the fields of cosmetics, medicine, and health products. In recent years, quinoa has been turned into a worldwide agricultural option, because of its extraordinary nutritional and industrial values, excellent adaptability and resistance to diverse climatic and soil conditions, such as abiotic factors of drought, salt, and cold (Feng et al., 2023; Park and Hahn, 2021). Quinoa edible grains are rich in many essential amino acids, especially lysine that is deficient in most cereals (Panuccio et al., 2014), and are free of gluten so as to make it a healthy substitute for celiac disease patients (Stikic et al., 2012). Additionally, quinoa grains also contain a high amount of other exceptionally nutriments, such as proteins, vitamins, fibers, minerals, iron, zinc, and calcium (Gordillo-Bastidas et al., 2016), and various bioactive substances including polyphenols, carotenoids, and oleic acid (Lan et al., 2023). However, quinoa seeds are achene type fruits with thin, dry and indehiscent pericarp, and the coat is formed by testa and tegument (Burrieza et al., 2014). This structure characteristic contributes to the increased permeability of testa, causing seeds easy to absorb water (Strenske et al., 2017). Furthermore, quinoa seed has a developed perisperm, the primary storage tissue that is full of hydrophilic starch granules (López-Fernández and Maldonado, 2013). It is precisely these reasons that affect quinoa seed moisture content, which in turn usually leads to seed poor storability during storage process and losing vigor more rapidly than other cereals. Therefore, maintaining seed vigor is of great importance for the long-term safe conservation of germplasm resources and the improvement of seed value in quinoa, and it is also an urgent issue that needs attention.

Seed vigor, a comprehensive index for evaluating several attributes of seed quality, reflects the potential for seed rapid germination and uniform emergence, which in turn influences normal seedling morphogenesis and field establishment under various environmental conditions (Souza et al., 2017). Seed vigor loss in quinoa, however, causes the declined seed quality and delayed germination, which has been a key factor restricting its production and application in modern agriculture (Souza et al., 2017). Seed vigor is determined by multiple factors, including genetic backgrounds, nutritional status and ecological conditions of parent plants, and storage conditions after harvest such as moisture content, storage temperature, and storage duration. It has been reported, in wheat (Chen et al., 2021), rice (Wang et al., 2022), and maize (Li et al., 2019), that there were great differences in vigor level and storability among seeds of different genotypes, indicating that genetic trait is the most key factor to affect seed vigor. Several studies on quinoa seed vigor mainly centered at its evaluation method based on artificial accelerated aging test (Souza et al., 2017), germination performance under different storage conditions (Strenske et al., 2017), and changes in seed storage proteins, amino acid profiles, and bioactive molecules (Aloisi et al., 2016). To date, however, the comprehensive response mechanisms of seed vigor loss induced by aging at the transcriptomic level are still unknown. Thus, elucidating the underlying mechanisms of seed vigor loss might provide greater insights into the breeding of new varieties with high vigor and long-term seed-safe conservation in germplasm bank. 

Seed vigor is a complex quantitative trait, which is closely related to many life events such as dormancy, germination, longevity, and storage. Therefore, it is very important to study the regulation mechanisms of seed vigor loss, such as degradation of nucleic acids and proteins, as well as lack of energy supply, so as to identify the genetic and physiological characteristics associated with seed vigor and enhance the ability of seeds to maintain alive during storage. So far, the genetic basis of seed vigor in wheat, rice, and maize has been extensively studied, and many quantitative trait loci and differentially expressed genes/abundant proteins (DEGs/DAPs) answerable for controlling seed vigor and storability have been identified (Chen et al., 2021; Li et al., 2019; Wang et al., 2022). However, the key genes and regulatory mechanisms involved in vigor loss of quinoa seeds during aging process have not yet been reported.

Previous studies, in *Arabidopsis*, rice, tobacco (*Nicotiana tabacum* L.), and chickpea (*Cicer arietinum* L.), have illustrated that multiple genes play vital roles in the regulation of increased or decreased seed vigor during storage (Narayana et al., 2017; Rissel et al., 2014; Verma et al., 2013; Yazdanpanah et al., 2019). These key genes contain small auxin-up RNA gene *OsSAUR33* (Zhao J. et al., 2021), isopropylmalate synthase *OsIPMS1* (He et al., 2019), aldehyde dehydrogenase *OsALDH7* (Shin et al., 2009), lipoxygenases *OsLOX2* and *LOX3* (Huang et al., 2014; Xu et al., 2015), peroxidases *PRX2* and *PRX25* (Renard et al., 2020), L-isoaspartyl methyltransferases *OsPIMT1* and *PIMT2* (Petla et al., 2016; Wei et al., 2015), galactinol synthases *CaGOS-1* and *CaGOS-2* (Salvi et al., 2016), and β-glucosidases *Os4BGlu14* (Ren et al., 2020). They contribute to regulating seed vigor mainly through functioning in energy metabolism, redox homeostasis, cellular detoxification, and macromolecular damage repair. Recently, several studies have also reported that seed vigor is regulated by *OsIAGLU* and bZIP23-PER1A module through mediating crosstalk of auxin and abscisic acid signaling pathway in rice (He et al., 2020; Wang et al., 2022). Although these above mentioned genes have been detected to control seed vigor, basically, they are functionally validated in rice seeds. However, whether there are new candidate genes to regulate seed vigor in quinoa are still unclear. Thus, it is highly necessary to reveal more key metabolic pathways and genes related to seed vigor loss, which will help further explore the novel possible mechanisms of seed vigor regulation in quinoa.

In the present study, quinoa seeds of Longli No.4 (L4) and Longli No.1 (L1) cultivars were exposed to artificial accelerated aging treatment under 75% relative humidity and 45°C, for durations of 0, 1, 2, and 4 d, respectively. Multispectral nondestructive testing and germination test were used to assess seed vigor differences. Furthermore, transcriptome sequencing was carried out to compare gene expression differences between two genotypes of seeds and obtain the key metabolic pathways and target genes controlling seed vigor. This study will provide data support and valuable insights into elucidating the regulatory mechanisms underlying quinoa seed vigor and storability.

## 2 Materials and methods

### 2.1 Seed materials

Two quinoa cultivars of Longli No.4 (L4) and Longli No.1 (L1) were used in this study, which were obtained from Animal Husbandry, Pasture and Green Agriculture Institute (Lanzhou, Gansu Province, China). From the appearance characteristics of seeds, they have different seed sizes and coat colors. Based on the preliminary experiment results, L4 cultivar was identified as the low seed vigor genotype with poor storage performance, while, L1 cultivar was confirmed as the high seed vigor genotype with good storage performance. After maturity and harvest, the plump seeds with uniform sizes were selected, and stored at -20°C in the airtight waterproof aluminum foil bags until artificial accelerated aging treatment.

### 2.2 Artificial accelerated aging treatment

Before artificial accelerated aging, L4 and L1 seeds were adjusted to the constant moisture content of 12% on fresh weight basis, according to saturated salt solution equilibrium static weighing method. For each cultivar, seed moisture content of at least three technical replicates were simultaneously adjusted and then mixed as one biological replicate. Next, seeds were placed in square boxes (110 × 110 × 20 mm, without the lid), which were then placed in a tightly closed plastic box (300 × 215 × 220 mm) with super-saturated NaCl solution at the bottom to obtain 75% relative humidity at 45°C. Seeds were exposed to the aging condition mentioned above for 0 (the control), 1, 2, and 4 d, respectively, which were defined as A0, A1, A2, and A4. After the specified aging duration, seeds were withdrawn immediately. For transcriptome sequencing and quantitative real-time PCR (qRT-PCR) analysis, the aged seeds were frozen in liquid nitrogen immediately and then stored at -80°C. For multispectral imaging analysis of seed vigor and germination test, the aged seeds were used within 1 h.

### 2.3 Multispectral imaging analysis of seed vigor

Multispectral imaging data acquisition was obtained by VideometerLab4 (Videometer A/S, Herlev, Denmark), which contained 19 different wavelengths (365, 405, 430, 450, 470, 490, 515, 540, 570, 590, 630, 645, 660, 690, 780, 850, 880, 940, and 970 nm) (Zhang et al., 2022). Totally, five replicates of approximate 100 seeds each were randomly taken from every seed batch (i.e. aging treatment) and placed in petri dishes, which were then placed on the bottom of spectrum instrument sphere, one after another. The multispectral raw images of seeds were obtained and manipulated by the Blob tool of VideometerLab software, with separating seeds from the irrelevant background. Morphological feature relevant data of each seed, including area (mm^2^), length (mm) and width (mm), color indexes, and saturation, were extracted and further analyzed. Normalized canonical discriminant analysis (nCDA), a supervised transformation construction method dividing multispectral images into regions of interest (ROI) with different spectral characteristics, is used for multivariate analysis of seed morphological information (Pornpanomchai et al., 2020). The nCDA can predict seed vigor with high accuracy, through marking and coloring the ROI based on the standardized spectral information of specified seed samples (Zhang et al., 2022). In this work, red and blue colors were used to standardize the multispectral images of A0 and A4 seeds, respectively, and the other images were transformed through nCDA analysis using MSI-Transformation Builder in Videometer software version 4.

### 2.4 Seed germination characteristics

Aged seeds of each quinoa cultivar, A0, A1, A2, and A4, were assayed for germination. Briefly, triplicates of 50 seeds each were placed on three layers of distilled water-moisturized filter papers in 110 × 110 mm petri dishes, which were then incubated at 25°C in the dark for 5 d. Seeds were considered as germinated when the radicle protruded through seed coat (at least 2 mm), and the germinated seeds were checked and recorded every 12 h. Seed vigor, including germination percentage (GP), germination index (GI), and vigor index (VI), were calculated.

### 2.5 Transcriptome sequencing

Total RNA of 24 samples (2 cultivars × 4 aging time points × 3 biological replicates) was extracted using Trizol reagent (Invitrogen, Carlsbad, CA, USA). The RNA purification, cDNA libraries construction, and sequencing by an Illumina HiSeq™ 4000 platform (San Diego, CA, USA) were performed as described earlier (Yan et al., 2022). After filtering reads with adapter pollution, more than 10% of unknown nucleotides (N), and more than 50% of low-quality (Q value ≤ 20) bases, the clean reads were mapped to quinoa reference genome (ASM168347v1, NCBI) using HISAT (version 2.1.0) (Kim et al., 2015). The *de novo* assembly and functional annotations of assembled unigenes were carried out according to the methods in previous publication (Yan et al., 2022).

### 2.6 Quantification of DEGs

Gene expression levels were calculated in terms of Fragments Per Kilobase of exon model per Million mapped fragments (FPKM) using RSEM software (version 1.1.11) (Li and Dewey, 2011). The DEGs were determined by DESeq2 software (version 1.22.1), with an adjusted *P*-value < 0.05 and fold change (FC) ≥ 2.0 (Love et al., 2014).

### 2.7 Temporal expression patterns analysis of DEGs

DEGs in L4 and L1 cultivars were subjected to unsupervised clustering using the fuzzy c-means algorithm in the Mfuzz package (Kumar and Futschik, 2007), to compare time-series gene expression profiles throughout the artificial accelerated aging durations of 0, 1, 2, and 4 d. The FPKM value of DEGs identified with an adjusted *P*-value < 0.05 were used for c-means clustering and the number of clusters was set to eight.

### 2.8 Functional enrichment analysis of DEGs

Gene Ontology (GO) enrichment and KEGG pathway analyses were performed through GO-seq R packages and KOBAS software, respectively, with a significant false discovery rate (FDR) < 0.05, to predict the potential biological functions of DEGs (Mao et al., 2005; Young et al., 2010).

### 2.9 Transcription factor analysis

Transcription factors (TFs) had activating or inhibiting effects on gene expression. The TF analysis was carried out using PlantTFDB 4.0 (version 4.0) (http://planttfdb.cbi.pku.edu.cn/), with a threshold of < e^-5^.

### 2.10 The qRT-PCR validation of DEGs

The same RNA samples for transcriptome sequencing analysis were reverse transcribed to cDNA using a Goldenstar™ RT6 cDNA Synthesis Kit Ver2 (TSK302S, Tsingke, Beijing, China). The qRT-PCR reaction system and program followed those which were described previously (Yan et al., 2022). Three biological replicates of each treatment were performed, and quinoa *Elongation Factor 1α* (*EF1α*) was used as the internal control gene. The relative expression levels of nine selected DEGs were calculated using 2^−ΔΔCt^ method on the basis of CT values (Livak and Schmittgen, 2001). The primers for qRT-PCR were designed and listed in **Supplementary Table S1**.

### 2.11 Statistical analysis

Data related to seed germination characteristics were expressed as means ± SE, and subjected to statistical analysis using SPSS software (SPSS Inc, version 17.0). Duncan’s test was used to evaluate difference among artificial accelerated aging durations, and Student’s t-test was used to compare difference between cultivars. Differences at the level of 5% and 1% were regarded as significant, which were labeled as * and **, respectively. To analyze the relationship between seed vigor and multispectral imaging, ‘candisc’ packages of R language (version 4.1) was used, with the default parameters.

## 3 Results

### 3.1 Comparison of morphological features and vigor levels of L4 and L1 seeds after artificial accelerated aging

**Figure 1:**
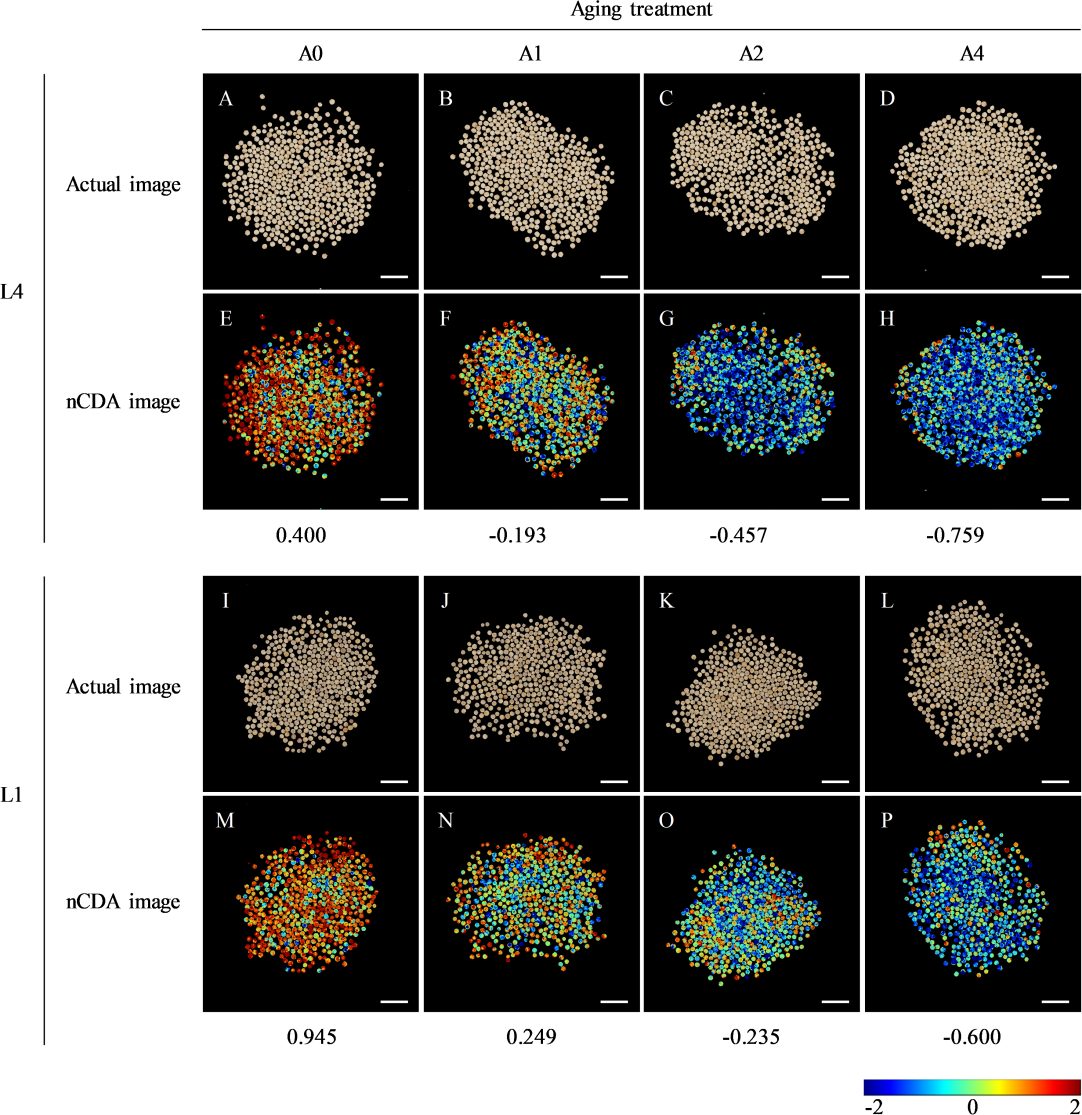
The RGB image and corresponding nCDA image of L4 and L1 quinoa seeds after artificial accelerated aging. **(A–D)** The RGB image and **(E–H)** corresponding nCDA image of L4 seeds at each aging time point. **(I–L)** The RGB image and **(M–P)** corresponding nCDA image of L1 seeds at each aging time point. Scale bar = 1 cm. A0, A1, A2, and A4 indicated the artificial accelerated aging duration of 0, 1, 2, and 4 d, respectively. In nCDA image, the deeper the red, the higher the seed vigor, while the darker the blue, the lower the seed vigor. Therefore, the more red seeds, the higher the vitality of seed batch, and the more blue seeds, the lower the vitality of seed batch.

Fourteen indexes of morphological features for L4 and L1 seeds after various aging durations were extracted by multispectral imaging and analyzed comparatively. The results showed that, in both L4 and L1 seeds, there were significant differences in five morphological indexes, through the paired comparative analysis of A1/A0, A2/A0, and A4/A0. Compared with unaged seeds, the three color related parameters, CIELab L*, CIELab A*, and CIELab B*, were all significantly increased in aged seeds. Also, the saturation and hue of aged seeds were significantly higher than those of unaged seeds. While, the shape related parameters of both L4 and L1 seeds, including area, length, width, and other indexes, did not show significant differences among various aging durations. In addition, differences in morphological features between two cultivars were analyzed. It was found that both shape indexes and color indexes between L4 and L1 seeds presented significant differences, at each aging duration (A0, A1, A2, and A4). In brief, based on the above analyses of seed morphological indicators, it indicated that L1 cultivar had significantly smaller seed size and darker seed coat color than L4 cultivar (**Figures 1A–D, I–L**; **Supplementary Table S2**).

Moreover, seed multispectral images were transformed through nCDA model, according to color standardization of high vigor (red) and low vigor (blue) seeds. The results showed that, as aging durations extend, the number of “red” seeds in both L4 and L1 cultivars decreased, while the number of seeds plotted in “blue” color increased (**Figures 1E–H, M–P**). Additionally, further analysis between two cultivars revealed that the nCDA value of L1 cultivar was higher than that of L4 cultivar at each aging time point, indicating that L1 cultivar had higher seed vigor level and storage resistance during artificial accelerated aging process.

### 3.2 Evaluation of seed germination characteristics of L4 and L1 cultivars after artificial accelerated aging

**Figure 2:**
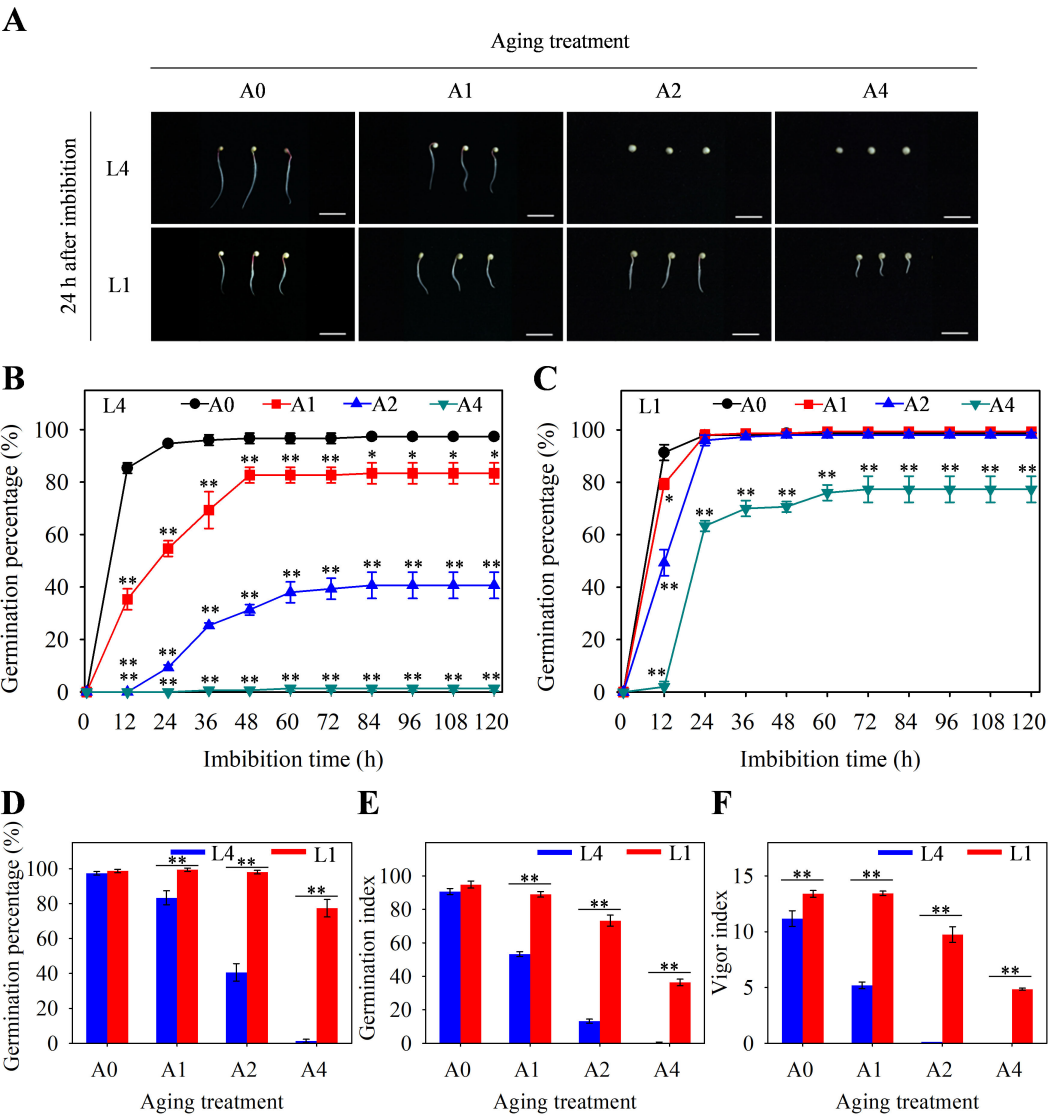
Germination characteristics of L4 and L1 quinoa seeds after artificial accelerated aging. **(A)** Phenotypic differences in germination after 24 h of seed imbibition. Scale bar = 1 cm. Germination percentage of **(B)** L4 and **(C)** L1 seeds during 120-hour imbibition process. **(D)** Germination percentage, **(E)** germination index, and **(F)** vigor index of L4 and L1 seeds after imbibition for 120 h. Values represent the means ± SE (n=3). Asterisk (*) in **(B)** and **(C)** means significant difference between aging group (A1, A2, and A4) and control group (A0), while asterisk (*) in **(D–F)** means significant difference between two cultivars of L4 and L1. The * and ** indicate the significant difference at 5% and 1% level, respectively, according to Student’s t-test.

Germination characteristics of aged seeds could reflect the vitality level and storability of quinoa seeds. It was found that the unaged seeds of both L4 and L1 could germinate rapidly, and generally, they germinated completely within 24 h after imbibition under normal condition (**Figures 2A–C**). As for aged seeds, germination phenotype evaluation revealed that the A2 and A4 seeds of L4 cultivar did not germinate, while those of L1 could still germinate normally within 24 h after imbibition, indicating that aging treatment resulted in reduced loss of seed vigor in L1 cultivar than in L4 cultivar (**Figure 2A**). Through further analyzing the germination changes during the 120-hour imbibition process, it showed that, in L4 cultivar, germination percentage of A1, A2, and A4 seed was significantly decreased, compared to unaged seeds (**Figure 2B**). However, in L1 cultivar, there were no significant differences between A1 and A2 aged seeds and unaged seeds, and only A4 aged seeds showed the significantly decreased germination percentage (**Figure 2C**). Moreover, comparative analysis of the final germination percentage after 120 h of imbibition revealed that this indicator was, respectively, 97% and 99% in unaged L4 and L1 seeds. However, after 4 d of aging treatment, it significantly decreased to 1% in L4 cultivar, but still remained at 77% in L1 cultivar (**Figure 2D**). In addition, germination index and vigor index of A1, A2, and A4 aged seeds were significantly higher in L1 cultivar than those in L4 cultivar (**Figures 2E, F**). Therefore, all these findings suggested that seeds of L1 cultivar were more storable than those of L4 cultivar during artificial accelerated aging process.

### 3.3 Transcriptome profiles involved in seed vigor responses of L4 and L1 after artificial accelerated aging

High-quality cDNA libraries of A0, A1, A2, and A4 seeds of L4 and L1 cultivars were prepared and sequenced using an Illumina HiSeq 4000 platform, to explore gene expression events involved in seed vigor responses and storability under artificial accelerated aging. After filtering the adapters and low-quality reads, a total of 39.05-50.67 million clean reads were generated from each sample, with an average of 46.12 million clean reads. The percentage of Q20 and Q30 bases were, respectively, 97.51%-98.06% and 93.26%-94.35%, and the GC content was 42.91%-45.17%, demonstrating that the original transcriptome data were of good quality and could be used for subsequent analyses. A ratio of 87.92%-96.64% clean reads was mapped to quinoa reference genome (ASM168347v1), with 83.43%-92.32% ones being uniquely mapped. The reads mapped to exon, intron, and intergenic regions were 92.90%-95.83%, 0.84%-2.23%, and 3.33%-5.43%, respectively (**Supplementary Table S3**). Furthermore, square of Pearson’s Correlation Coefficient (R^2^) was used to evaluate the correlation of biological repetitions, and it showed that the repetitive values between samples ranged among 0.90-1.00, indicating the high repeatability of dataset (**Supplementary Figure S1**).

**Figure 3:**
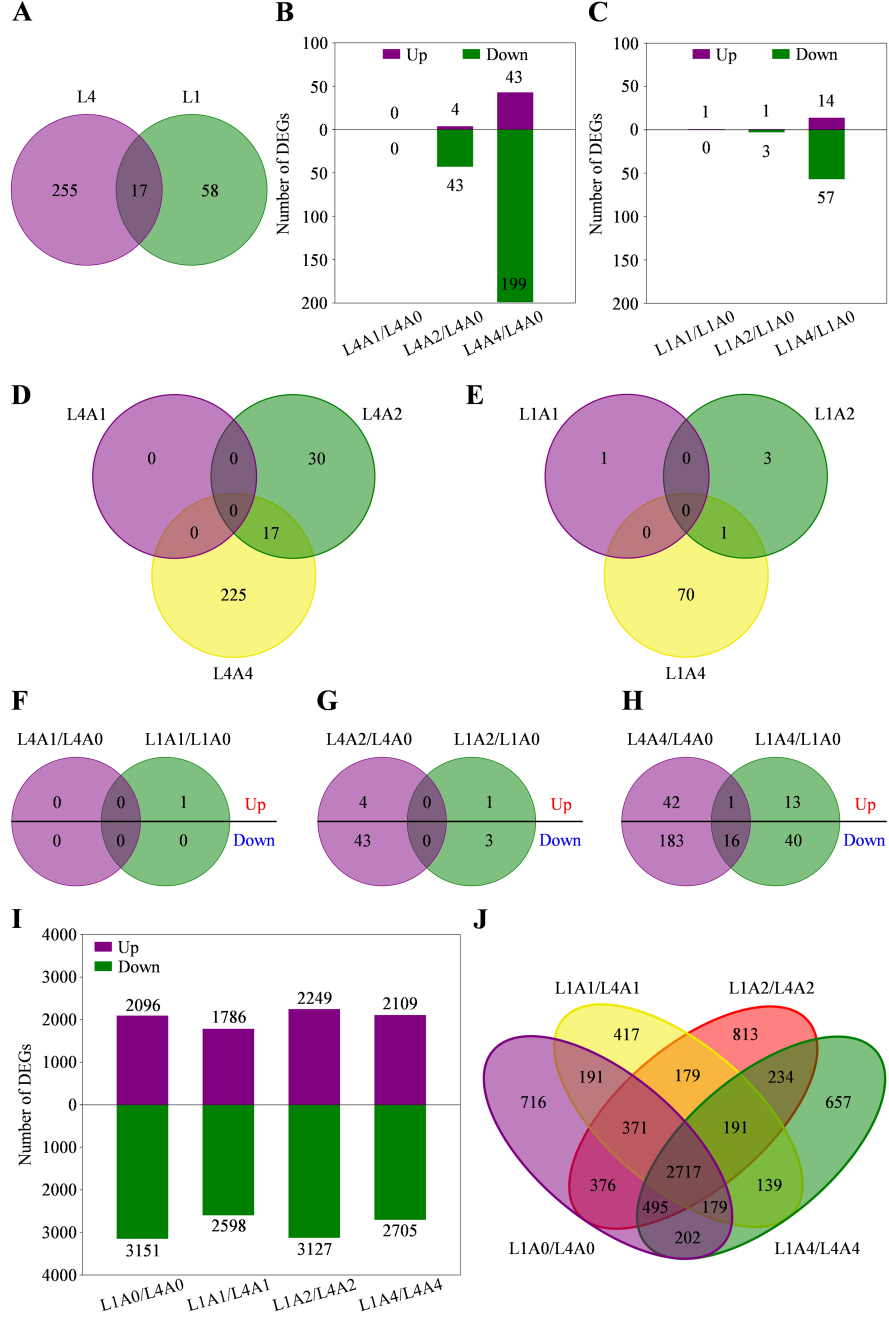
Differentially expressed genes (DEGs) related to seed vigor responses of L4 and L1 cultivars under artificial accelerated aging. **(A)** Venn diagram of DEGs between L4 and L1. **(B, C)** Number of DEGs in L4 and L1 cultivars, respectively, in pairwise comparison of aging group and control group. **(D, E)** Venn diagrams of DEGs among different aging durations in L4 and L1 cultivar, respectively. **(F–H)** Venn diagrams of up- and down-regulated DEGs between two cultivars after aging for 1, 2, and 4 d, respectively. **(I)** Number of DEGs in pairwise comparison of L1/L4 at different aging durations. **(J)** Venn diagram of DEGs between two cultivars at different aging durations.

Gene expression level of each sample was calculated using FPKM, and DEGs were identified based on the selection criteria of |log2FC| ≥ 1 and adjusted *P*-value < 0.05. Totally, 272 and 75 DEGs were, respectively, identified in L4 and L1 seeds after aging treatment, among which 17 ones were shared (**Figure 3A**). Specifically, in L4 cultivar, 0, 47 (4 up- and 43 down-regulated), and 242 (43 up- and 199 down-regulated) DEGs were, respectively, identified in A1/A0, A2/A0, and A4/A0; while in L1 cultivar, 1 (up-regulated), 4 (1 up- and 3 down-regulated), and 71 (14 up- and 57 down-regulated) DEGs were respectively identified (**Figures 3B, C**; **Supplementary Table S4**). Similarly, there were no common DEGs detected in both cultivars among the above aging durations (**Figures 3D, E**). Hence, DEGs expression relationships between two cultivars at each aging time node were further analyzed. It showed that no shared DEGs were detected at A1 and A2 nodes, and only 17 (1 up- and 16 down-regulated) common ones were identified at A4 node (**Figures 3F–H**; **Supplementary Table S4**). In addition, to reveal the differences in gene responses between genotypes under artificial accelerated aging, the expression analysis of cultivar-specific genes was performed. It was found that a total of 5247 (2096 up- and 3151 down-regulated), 4384 (1786 up- and 2598 down-regulated), 5376 (2249 up- and 3127 down-regulated), and 4814 (2109 up- and 2705 down-regulated) DEGs were identified, respectively, through pairwise comparison of L1/L4 at A0, A1, A2, and A4 nodes (**Figure 3I**). Among the above DEGs, 716, 417, 813, and 657 ones were exclusively included at A0, A1, A2, and A4, respectively; while 2717 ones were simultaneously detected at all aging durations, indicating that these DEGs were specific and common in response to artificial accelerated aging in L1 cultivar (**Figure 3J**).

**Figure 4:**
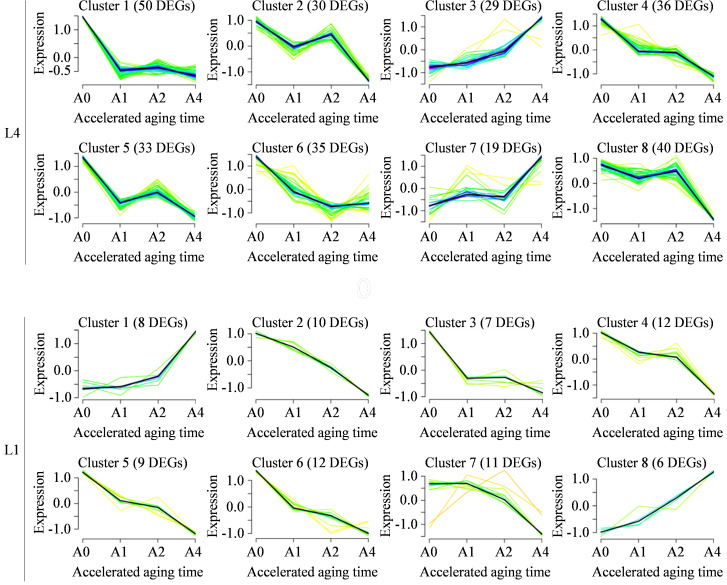
Time-series expression patterns analysis of DEGs in L4 and L1 cultivars across four aging durations determined using Mfuzz. Each diagram illustrates a type of expression profile of dynamic changes in DEGs, with the order of clusters and the number of DEGs in the upper part.

Afterwards, the time-series expression patterns of DEGs in L4 and L1 cultivars were clustered using Mfuzz package, to gain the deeper insight into dynamic expression changes of genes associated with seed vigor under artificial accelerated aging (**Figure 4**). For L4 cultivar, 272 DEGs were assigned into eight clusters, including two upregulated profile models, cluster 3 and 7 (29 and 19 DEGs, respectively), and six downregulated profile models, cluster 1, 2, 4, 5, 6, and 8 (50, 30, 36, 33, 35, and 40 DEGs, respectively). Similarly, 75 DEGs in L1 cultivar were also assigned into eight clusters, including two upregulated patterns of cluster 1 and 8 that contained eight and six DEGs, and six downregulated patterns of cluster 2-7 that had 10, seven, 12, nine, 12, and 11 DEGs.

### 3.4 GO enrichment and KEGG pathway analyses of DEGs involved in seed vigor responses of L4 and L1 after artificial accelerated aging

To describe the gene properties and biological functions related to seed vigor responses under artificial accelerated aging, GO enrichment analysis of DEGs was performed by annotating them into three major categories, including biological process (BP), cellular component (CC), and molecular function (MF). Based on the GO categories of aging duration-specific DEGs detected in pairwise comparisons of A1/A0, A2/A0, and A4/A0, a total of zero, 33, 22 significant GO terms in L4 and zero, 47, one significant GO terms in L1 were enriched, respectively. Moreover, according to the GO categories of cultivar-specific DEGs, it was found that 71, 56, 63, and 35 GO terms were significantly enriched in pairwise comparison of L1/L4 at A0, A1, A2, and A4 points, respectively (**Supplementary Table S5**). These findings indicated that there were more GO terms enriched by cultivar-specific DEGs than by aging duration-specific DEGs. Therefore, the significantly enriched GO terms of cultivar-specific DEGs were further analyzed at different aging durations, with a threshold of FDR < 0.05. Top 20 significantly enriched GO terms showed that DEGs were involved in the important terms associated with heat stress responses in seeds, such as response to heat, oxidoreductase activity, dioxygenase activity, carboxylic acid binding, organic acid binding, hydrolase activity, L-ascorbic acid binding, cysteine synthase activity, etc. (**Supplementary Figure S2**).

**Figure 5:**
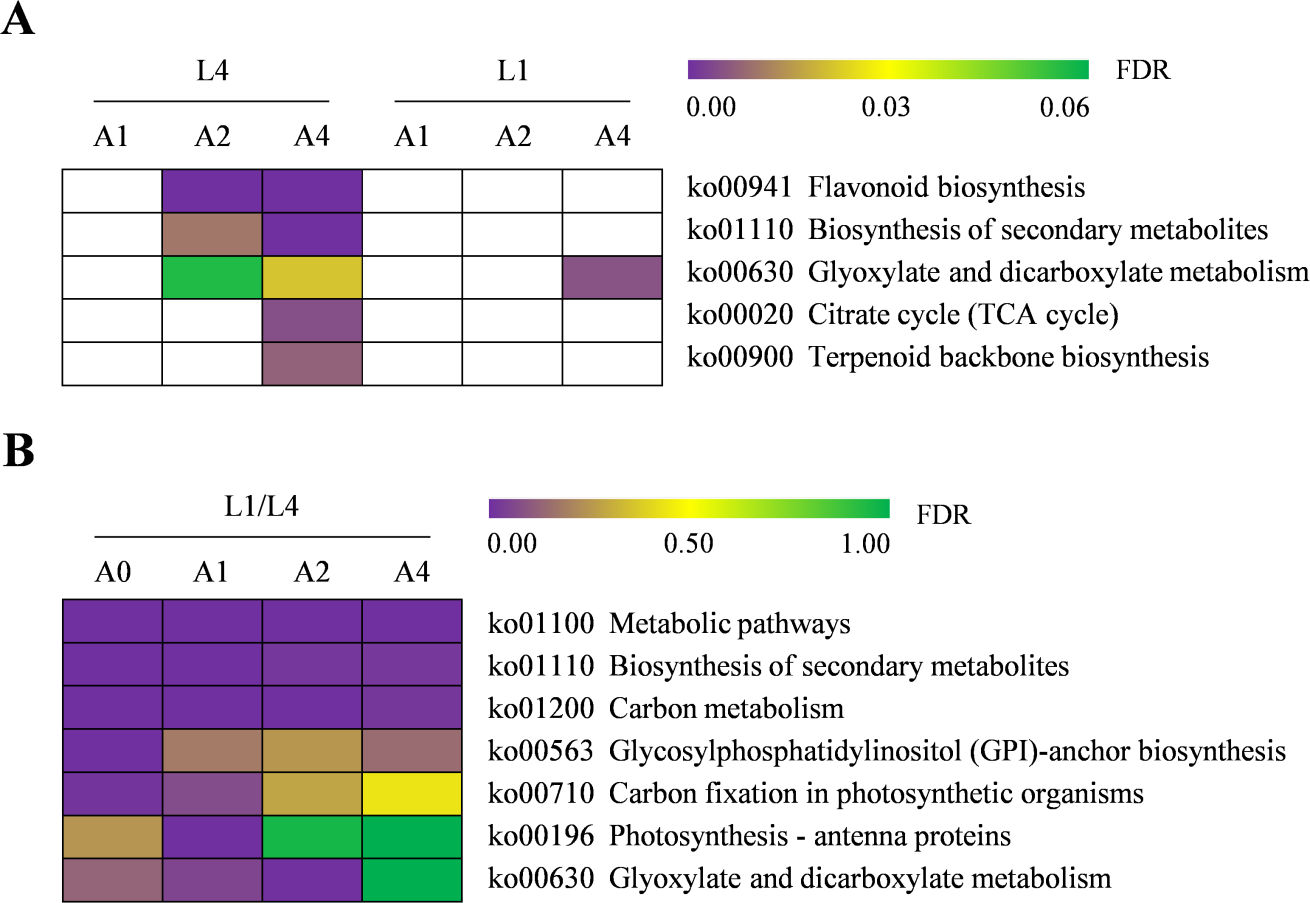
Heatmap of significantly enriched KEGG pathways of DEGs in L4 and L1 seeds under artificial accelerated aging. **(A)** Pairwise comparisons of various aging durations in each cultivar. The white color block indicated the pathways that were not enriched. **(B)** Pairwise comparison of two cultivars at various aging durations. The gene ID involved in each pathway is detailed in **Supplementary Table S6**.

To characterize the molecular functions of DEGs involved in seed vigor responses in two cultivars, KEGG pathway analysis was carried out. The results analyzed from aging duration-specific DEGs in pairwise comparisons of A1/A0, A2/A0, and A4/A0 revealed that a total of zero, 25, 76 pathways in L4 and zero, zero, 28 pathways in L1 were enriched, respectively. However, for the cultivar-specific DEGs, 134, 131, 136, and 133 KEGG pathways were enriched at A0, A1, A2, and A4, respectively (**Supplementary Table S6**). Considering the important roles of pathways, the significantly enriched ones were further focused on, with a threshold of FDR < 0.05. After artificial accelerated aging, there were no significantly enriched pathways in L4 seeds at A1 and in L1 seeds at A1 and A2. However, with aging duration prolonging, the pathways of flavonoid biosynthesis, biosynthesis of secondary metabolites, glyoxylate and dicarboxylate metabolism, citrate cycle (TCA cycle), and terpenoid backbone biosynthesis were significantly enriched in L4, among which glyoxylate and dicarboxylate metabolism was simultaneously enriched in L1 cultivar at A4 (**Figure 5A**). Therefore, the significantly enriched pathways only in L4 cultivar might be the most critical ones affected by artificial accelerated aging, which might also be closely related to the low seed vigor of L4 genotype.

Additionally, for cultivar-specific DEGs, the KEGG pathways of metabolic pathways, biosynthesis of secondary metabolites, and carbon metabolism were enriched significantly at all aging durations, indicating that these pathways might play important roles in seed storage tolerance of high vigor genotype. Furthermore, several other pathways were also enriched significantly, such as glycosylphosphatidylinositol (GPI)-anchor biosynthesis, carbon fixation in photosynthetic organisms, and glyoxylate and dicarboxylate metabolism (**Figure 5B**). Thus, taking into account the above results, DEGs involved in the key pathways of flavonoid biosynthesis, TCA cycle, terpenoid backbone biosynthesis, and carbon metabolism were further analyzed.

### 3.5 DEGs involved in the significantly enriched KEGG pathways associated with seed vigor of quinoa under artificial accelerated aging

To uncover the key DEGs related to seed vigor of quinoa under artificial accelerated aging, two types of KEGG pathways were focused on. Namely, one type was the pathways significantly enriched only in L4 through pairwise comparisons of aging durations in each cultivar, and the other type was the ones significantly enriched in pairwise comparison of L1/L4 at all aging durations. These two types of pathways, respectively, revealed the important DEGs that were closely related to low seed vigor of L4 genotype and high seed vigor of L1 genotype.

**Figure 6:**
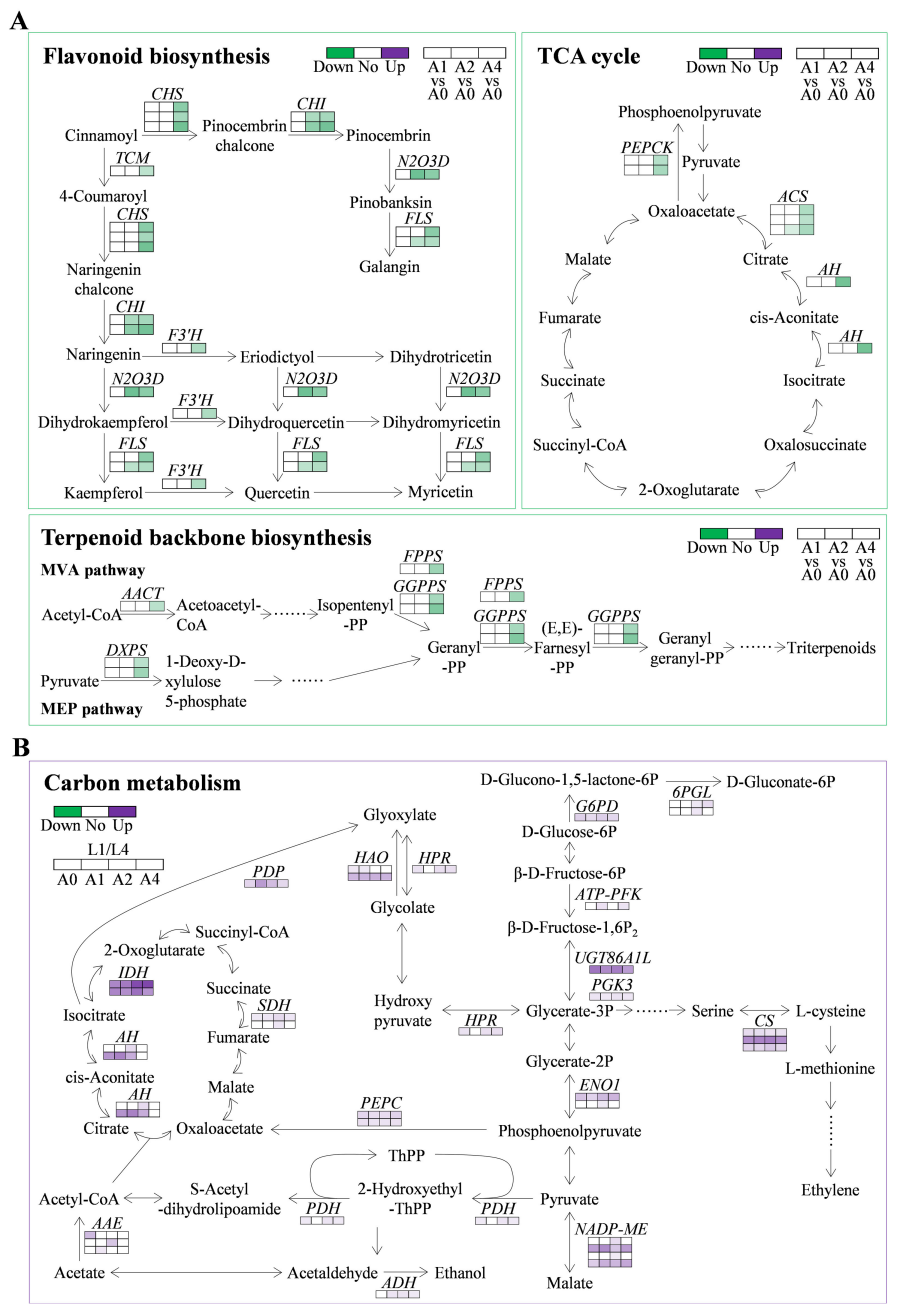
Expression changes of DEGs involved in the significantly enriched KEGG pathways in L4 and L1 seeds under artificial accelerated aging. **(A)** DEGs in flavonoid biosynthesis, citrate cycle (TCA cycle), and terpenoid backbone biosynthesis in low seed vigor genotype under artificial accelerated aging. **(B)** DEGs in carbon metabolism pathway in pairwise comparison of L1/L4 at all aging durations. CHS, chalcone synthase; CHI, chalcone isomerase; TCM, trans-cinnamate 4-monooxygenase; N2O3D, naringenin, 2-oxoglutarate 3-dioxygenase; FLS, flavonol synthase; F3’H, flavonoid 3’-monooxygenase; AACT, acetyl-CoA acetyltransferase; DXPS, probable 1-deoxy-D-xylulose-5-phosphate synthase; FPPS, farnesyl pyrophosphate synthase; GGPPS, geranylgeranyl pyrophosphate synthase; G6PD, glucose-6-phosphate dehydrogenase; 6PGL, 6-phosphogluconolactonase; ATP-PFK, ATP-dependent 6-phosphofructokinase 5; UGT86A1L, UDP-glycosyltransferase 86A1-like; PGK3, phosphoglycerate kinase 3; ENO1, enolase 1; PDH, pyruvate dehydrogenase E1 component; ADH, alcohol dehydrogenase; AAE, acetate/butyrate-CoA ligase; AH, aconitate hydratase; IDH, isocitrate dehydrogenase; SDH, succinate dehydrogenase; PEPC, phosphoenolpyruvate carboxylase; NADP-ME, NADP-dependent malic enzyme; HPR, glyoxylate/hydroxypyruvate reductase; CS, cysteine synthase; PDP, petal death protein; HAO, hydroxyacid oxidase.

Three significantly enriched KEGG pathways were involved in low seed vigor of L4 cultivar, including flavonoid biosynthesis, citrate cycle (TCA cycle), and terpenoid backbone biosynthesis (**Figure 5A**). Totally, 14, six, and six DEGs were detected in these three pathways, respectively (**Supplementary Table S7**). Interestingly, it showed that all the identified DEGs were downregulated, especially when aging duration was extended to 4 days, with a decrease of 0.22-0.49 folds (**Figure 6A**; **Supplementary Table S7**). The results indicated that DEGs in the above three pathways might be key factors leading to the low seed vigor of L4 cultivar under artificial aging conditions.

Moreover, it was found that carbon metabolism was enriched significantly in pairwise comparison of L1/L4 at all aging durations (**Figure 5B**), which might play the key role in high seed vigor of L1 cultivar. A total of 36 upregulated DEGs were detected, among which 13, six, three, one, three, one, six, and three were, respectively, involved in glycolysis/gluconeogenesis (ko00010), citrate cycle (TCA cycle, ko00020), pentose phosphate pathway (ko00030), glycine, serine and threonine metabolism (ko00260), cysteine and methionine metabolism (ko00270), valine, leucine and isoleucine degradation (ko00280), pyruvate metabolism (ko00620), and glyoxylate and dicarboxylate metabolism (ko00630) (**Figure 6B**). It was worth noting that 18 DEGs were upregulated simultaneously at all aging durations, and in particular, *UDP-glycosyltransferase 86A1-like* (*UGT86A1L*, LOC110687215), *enolase 1* (*ENO1*, novel.788), *isocitrate dehydrogenase* (*IDH*, LOC110720542 and LOC110740096), *NADP-dependent malic enzyme* (*NADP-ME*, LOC110696234 and LOC110707699), *hydroxyacid oxidase 2-like* (*HAO2L*, LOC110722404), *cysteine synthase 2-like* (*CS2L*, LOC110735467), and *methylmalonate-semialdehyde dehydrogenase* (*MSDH*, LOC110740061) were upregulated by 20.46-65.88, 3.79-9.62, 37.52-258.92 (26.24-76.05), 5.77-32.82 (4.56-14.09), 8.56-10.62, 29.71-49.20, and 6.45-13.14 folds, respectively (**Supplementary Table S7**). The results indicated that the upregulated DEGs in carbon metabolism acted important roles in resisting loss of seed vigor in high-vigor genotype under artificial accelerated aging.

### 3.6 Transcription factors involved in seed vigor responses of L4 and L1 after artificial accelerated aging

TFs prediction and statistical analysis of the specific and common DEGs in L4 and L1 cultivars were carried out using PlantTFDB 4.0. Totally, 18 L4-specific and three L1-specific TFs were identified. BH130, WRKY23, and TCP22 were all downregulated and specifically expressed in L4 cultivar, whereas TFs including MADS-box protein JOINTLESS-like and protein FAR1-related sequence 5-like were differentially expressed and uniquely found in L1 cultivar (**Supplementary Table S8**). For specific TFs, the top three representative families with the most members were bHLH (ten members), NAC (eight members), and WRKY (seven members); while, the most typical representative families for common TFs were bHLH, FAR1, and AP2/ERF-ERF, with each having ten members (**Table 1**). During aging process, most TF families were downregulated and differed in the numbers. The bZIP family was enriched in the common DEGs, which was previously reported in regulating seed vigor. Furthermore, the NAC, WRKY, B3, MYB, and bHLH families were multifunctional and played important roles in response to abiotic stress defense.

**Table 1:** Specific and common TFs involved in different aging durations in L1 cultivar, compared to L4 cultivar.

Specific TFs in L1				Common TFs in L4 and L1			
TF family	Total	Up	Down	TF family	Total	Up	Down
bHLH	10	2	8	bHLH	10	1	9
NAC	8	3	5	FAR1	10	3	7
WRKY	7	3	4	AP2/ERF-ERF	10	5	5
C2H2	6	1	5	B3	6	4	2
GARP-G2-like	6	2	4	bZIP	5	1	4
MADS-MIKC	6	2	4	C2H2	4	1	3
DBB	6	0	6	GNAT	4	2	2
MYB	5	1	4	AP2/ERF-AP2	4	2	2
FAR1	4	1	3	HB-HD-ZIP	4	2	2
B3	4	2	2	NAC	3	0	3
GNAT	4	3	1	MYB	3	2	1
LOB	4	1	3	AUX/IAA	3	2	1
Trihelix	4	1	3	C2C2-CO-like	3	0	3
AP2/ERF-ERF	3	0	3	HSF	3	0	3
TRAF	3	1	2	MADS-M-type	3	1	2

### 3.7 The qRT-PCR validation of DEGs involved in seed vigor responses after artificial accelerated aging

To confirm the accuracy of transcriptome results, qRT-PCR analysis was performed using the same RNA-seq samples. A total of nine DEGs involved in the significantly enriched pathways of flavonoid biosynthesis, TCA cycle, terpenoid backbone biosynthesis, and carbon metabolism were selected, including five downregulated ones (*F3’5’MTL*, *flavonoid 3’,5’-methyltransferase-like*; *CHI3*, *probable chalcone-flavonone isomerase 3*; *F3HL*, *flavonol synthase/flavanone 3-hydroxylase-like*; *ACSα1L*, *ATP-citrate synthase alpha chain protein 1-like*; and *FPPS1L*, *farnesyl pyrophosphate synthase 1-like*) and four upregulated ones (two *IDH*, *NADP-ME*, and *HAO2L*). Interestingly, the results showed that expression profiles of almost all selected DEGs by qRT-PCR were in accordance with the transcriptome data (**Supplementary Figure S3**), which confirmed the reliability of RNA-seq analyses.

## 4 Discussion

Seed vigor is an important indicator of seed quality, which affects the germination under favorable or adverse environmental conditions, and determines seed longevity and long-term safe conservation in germplasm bank (Lee et al., 2019). Seeds gradually lose their vigor during storage, and artificial accelerated aging method has been widely used for rapid and effective evaluation of seed vigor in several crops (Delouche and Baskin, 1973; Fenollosa et al., 2020). Artificial accelerated aging test is a simulation method for seed storage and vigor evaluation, which is based on the increased deterioration rates when seeds are exposed to high temperature and high relative humidity. However, it is limited to species with large seeds, and differences in small-seeded species such as quinoa may affect the consistency of results due to the difficulty and unevenness of seed moisture control (Powell, 1995). In quinoa, the unique seed structural characteristics result in high permeability of seed coat, which makes seeds prone to absorbing water and reducing vigor and quality (Burrieza et al., 2014; Strenske et al., 2017; López-Fernández and Maldonado, 2013). Therefore, a possible strategy to address this problem is to use saturated salt solutions with the purpose of declining the relative humidity inside the sealed container where seeds are placed in (Souza et al., 2017). In recent years, great progress has been made, in traditional crop species such as wheat, rice, and maize, to reveal the molecular mechanisms of seed vigor regulation under natural storage or artificial aging (Chen et al., 2021; Li et al., 2019; Wang et al., 2022). However, the underlying mechanisms of seed vigor responses to aging in the promising crop of quinoa are still obscure. In this study, the difference of seed vigor between two quinoa cultivars of L4 and L1 was identified through multispectral imaging analysis and germination test, and it was found that seed vigor of L1 cultivar was superior to that of L4 cultivar (**Figures 1**, **2**). Next, in order to explore the involved molecular mechanisms, a comparative transcriptome analysis was carried out on the artificially aged seeds of these two different cultivars, and it revealed that a large number of genes were significantly altered in expression (**Figure 3**; **Supplementary Table S4**). Further, the significantly and specifically enriched pathways were identified, including flavonoid biosynthesis, TCA cycle, and terpenoid backbone biosynthesis in L4 seeds and carbon metabolism in L1 seeds, suggesting that these pathways, respectively, might be closely related to the low seed vigor of L4 and high seed vigor of L1 during artificial aging (**Figures 5**, **6**; **Supplementary Tables S6**, **S7**).

### 4.1 Storage sensitive seeds possess a weaker flavonoid biosynthesis

Flavonoids, the widely present secondary metabolites in plants, are key participants in plant growth and resistance to stresses (Wu et al., 2023). On one hand, flavonoids play a vital role in regulating root growth and plant color (Franco et al., 2015; Xiao et al., 2022). On the other hand, flavonoids enhance plant resistance to various abiotic and biotic stresses, such as UV-B (Gu et al., 2022), drought (Li et al., 2021), heavy metal (Sharma et al., 2021), cold (Xie et al., 2018), insects (Aslam et al., 2022), and fungal pathogens (Irani et al., 2018), through removing reactive oxygen species (ROS), increasing antioxidant activity, promoting photosynthesis, influencing stomatal activity, and restoring protein accumulation and bioavailability (Neugart and Schreiner, 2018; Peng et al., 2022). In addition, flavonoids are considered to have an essential function in seed vigor and longevity, since previous research in rice indicated that a positive correlation of seed vigor with flavonoid content was observed, which confirmed the superiority of seed vigor in pigmented rice over non-pigmented rice (Sahoo et al., 2020). In this study, based on the multispectral phenotypic analysis of L4 and L1 quinoa seeds, it was found that color related indicators presented significant differences (**Figures 1A–D, I–L**; **Supplementary Table S2**), indicating that L1 cultivar had significantly darker seed coat color than L4 cultivar with or without exposing to aging. Meanwhile, germination test results showed that germination phenotype, germination percentage, germination index, and vigor index of L1 aged seeds were significantly better than those of L4 aged seeds (**Figure 2**), suggesting L1 seeds were more storable and advantageous to preventing seed vigor loss than L4 seeds so as to maintain their high level when subjected to aging conditions. In seeds, the structural diversity of flavonoids and its accumulation are reported to have a significant impact on seed color, and, generally, the higher the flavonoid content, the darker the seed color (Galland et al., 2014; Guo et al., 2022). The seed quality traits in pigmented rice genotypes such as germination, seed vigor index, and total flavonoid content were found superior, which revealed the importance of pigmentation due to flavonoids accumulation for improving seed quality (Jena et al., 2022). There was more direct evidence to report that flavonoids in the pericarp of pigmented rice seed played a significant role in seed longevity by reducing oxidation occurring during storage (Righetti et al., 2015). However, after exposure to storage environment or aging condition, flavonoids that acted as protectants in seeds could degrade, resulting in loss of seed vigor and poor germination (Ali et al., 2018; Awolu et al., 2022). According to the KEGG enrichment analysis in transcriptome, it was found that the flavonoid biosynthesis pathway in L4 seeds was significantly enriched and the involved genes were significantly downregulated during aging process, while this pathway in L1 seeds was not significantly enriched (**Figures 5A**, **6A**). Therefore, it was speculated that the degree of seed vigor loss in L4 cultivar was greater than that in L1 cultivar, which might be closely related to the excessive degradation of flavonoids in L4 seeds.

As a large family and the best-studied secondary metabolites in plants, flavonoids are categorized into several classes including flavones, flavonols, dihydroflavones, dihydroflavonols, isoflavones, dihydroisoflavones, chalcones, anthocyanins, and proanthocyanidins (Galland et al., 2014). It is proven that plants accumulate flavonoids under stresses by regulating the expression of genes related to flavonoid synthesis (Wu et al., 2023). In *Arabidopsis*, a set of mutants (*transparent testa*, *tt*) impaired in flavonoid biosynthesis pathway presented a reduced seed longevity upon aging (Debeaujon et al., 2000; Niñoles et al., 2023). However, it also reported that *chalcone synthase* (*CHS*) and *chalcone isomerase* (*CHI*) genes were, respectively, able to complement their orthologous defect in *Arabidopsis tt4* and *tt5* mutants (Shih et al., 2008), indicating that these genes compensated for the unfavorable phenotype by regulating flavonoid synthesis. CHS, a polyketide synthase, is the first rate-limiting enzyme in flavonoid biosynthesis pathway (Zhao C. et al., 2021). Then, CHI catalyzes the second step and guarantees the rapid formation of biologically active (S)-isomer (Yin et al., 2019). Generally, CHIs are classified into two types in plants, and type I is responsible for the conversion of naringenin chalcone into naringenin (Yin et al., 2019). Kaempferol is a natural flavonol showing antioxidant property and exists in many plants, such as vegetables, fruits, and herbs. It is produced under the catalysis of naringenin, 2-oxoglutarate 3-dioxygenase (N2O3D) and flavonol synthase (FLS). N2O3D catalyzes the reaction of naringenin, eriodictyol, and dihydrotricetin to respectively produce dihydrokaempferol (DHK), dihydroquercetin (DHQ), and dihydromyricetin (DHM). FLS is a FeII/2-oxoglutarate-dependent dioxygenase and catalyzes the desaturation of dihydroflavonols to produce flavonol, namely, DHK, DHQ, and DHM are respectively converted to kaempferol, quercetin, and myricetin (Meng et al., 2019). Whereas flavonoid 3’-monooxygenase (F3’H) causes the degradation of naringenin to eriodictyol, DHK to DHQ, and kaempferol to quercetin (Kumari et al., 2023). Herein, transcriptome data revealed that genes involved in flavonoids biosynthesis, *CHS*, *CHI*, *N2O3D*, *FLS*, and *F3’H*, were downregulated in L4 quinoa seeds during aging, while this pathway was not significantly enriched in L1 seeds (**Figures 5A**, **6A**). These findings indicated that the adverse effects of aging treatment on flavonoids biosynthesis in storage sensitive seeds were more serious, thus leading to a more severe seed vigor loss and germination inhibition.

### 4.2 Storage sensitive seeds have an insufficient ability of terpenoid backbone biosynthesis

Terpenoids are important secondary metabolites in plants, and many of them have good antioxidant properties (Sun et al., 2023). Therefore, they are believed to protect plants from various abiotic and biotic stresses, for instance, oxidative damage, temperature, drought, and even pests. Based on the free radical theory of seed aging, it reports that the excessive accumulated ROS are considered to induce the structural degradation and functional deterioration of macromolecular substances, including membrane lipids, proteins and nucleic acids (Kurek et al., 2019). Generally, terpenoids in plants are biosynthesized from a C5 isopentenyl diphosphate unit through two independent pathways, namely, the mevalonic acid (MVA) pathway and the methylerythritol phosphate (MEP) pathway (Vranová et al., 2013). There are a series of branch-points enzymes, such as acetyl-CoA acetyltransferase (AACT), 1-deoxy-D-xylulose-5-phosphate synthase (DXPS), farnesyl pyrophosphate synthase (FPPS), and geranylgeranyl pyrophosphate synthase (GGPPS), which are involved in the above two pathways and catalyze the formation of carbocyclic skeleton of terpenoids (Nagegowda and Gupta, 2020). AACT, also called acetoacetyl-CoA thiolase, catalyzes hydrolysis and condensation of the first enzymatic step in MVA biosynthesis pathway. It converts two acetyl-CoA to produce acetoacetyl-CoA and is an essential regulatory enzyme for terpenoid backbone biosynthesis under abiotic stress (Soto et al., 2011). However, DXPS is the key enzyme in the MEP biosynthesis pathway of terpenoid, catalyzing the first and rate-limiting step in the formation of 1-deoxy-D-xylulose 5-phosphate from pyruvate (Tian et al., 2023). Next, FPPS catalyzes the consecutive condensations of dimethylallyl pyrophosphate or geranyl pyrophosphate to form farnesyl pyrophosphate, which is an important precursor for the biosynthesis of terpenoids, for example, polyisoprenoids in plants that produce natural rubber (Wu et al., 2017). Further, GGPPS catalyzes the condensation of dimethylallyl pyrophosphate, isopentenyl pyrophosphate and geranyl pyrophosphate to form geranylgeranyl pyrophosphate (Zhang et al., 2021). So far, many genes involved in terpenoid biosynthesis have been identified in plant species, such as *Arabidopsis thaliana* (Lange and Ghassemian, 2003), *Cassia tora* (Tian et al., 2023), *Hevea brasiliensis* (Wu et al., 2017), and *Liriodendron tulipifera* (Zhang et al., 2021). In quinoa, it has been reported that terpenoids accumulation varied in tissue-specific patterns and deposited in the seed coat (Tabatabaei et al., 2022). In the present study, the KEGG pathway of terpenoid backbone biosynthesis was significantly enriched in L4 seeds after aging treatment, and the involved genes including *AACT*, *DXPS*, *FPPS*, and *GGPPS* were significantly downregulated. However, terpenoid backbone biosynthesis was not significantly enriched in L1 seeds (**Figures 5A**, **6A**). Therefore, the results suggested that the impact on terpenoids induced by aging might be a significant reason for more vigor loss in storage sensitive seeds, compared to storage tolerant seeds.

### 4.3 Storage tolerant seeds maintain carbon metabolism and energy supply

Carbon metabolism is composed of the tricarboxylic acid (TCA) cycle, glycolysis, as well as metabolisms of sugars, polyols, organic acids, and fatty acids (Zhang et al., 2018). Under stress conditions, it is maintained by multiple regulatory levels in cells to resist the adverse damages (Santos-Filho et al., 2014). Pentose phosphate pathway (PPP) is a pivotal sugar metabolism route widely distributed in plants (Cardi et al., 2016). It can provide carbon skeleton and NADPH for the synthesis of fatty acids during seed development, further playing important roles in seed germination and seed vigor (Xue et al., 2017). The PPP starts with two irreversible steps that are catalyzed by glucose-6-phosphate dehydrogenase (G6PD) and 6-phosphogluconolactonase (6PGL), to convert glucose-6-phosphate to 6-phosphogluconolactone (Lansing et al., 2020; Xiong et al., 2009). Xin et al. (2011) found that the PPP was inhibited during aging process of maize seeds and the specific activity of G6PD was also reduced, suggesting the important roles of stored carbohydrate mobilization and energy supply in seed aging and seed vigor. In this study, *G6PD* and *6PGL* were upregulated in L1 seeds, indicating that PPP might be activated by artificial aging and prepare for promoting carbohydrate mobilization and energy supply (**Figure 6B**).

Glycolysis is a metabolic pathway necessary for mobilizing stored substances (Dong et al., 2015). It provides pyruvate to the TCA cycle and mitochondrial respiration for further ATP production (Zhao and Assmann, 2011; Chang et al., 2017). In this study, several genes involved in glycolysis were upregulated in L1 seeds, such as ATP-dependent 6-phosphofructokinase 5 (*ATP-PFK*), UDP-glycosyltransferase 86A1-like (*UGT86A1L*), and *phosphoglycerate kinase 3* (*PGK3*), suggesting that these genes might exert positive effects on carbohydrate metabolism and energy supply in storage tolerant seeds of quinoa (**Figure 6B**). Phosphoenolpyruvate (PEP) is a central intermediate in the metabolism of prokaryotes and eukaryotes (Prabhakar et al., 2009). Previous research in aged oat (*Avena sativa* L.) seeds had reported that melatonin promoted the accumulation of PEP to accelerate germination (Yan and Mao, 2021). Herein, *enolase 1* (*ENO1*) involved in PEP production was upregulated in L1 seeds, indicating that PEP participated in the energy supply of storage tolerant seeds of quinoa (**Figure 6B**). Pyruvate acts as the final product of glycolysis and is further converted into acetyl-CoA, an important intermediate in TCA cycle, through the catalysis of pyruvate dehydrogenase E1 component (PDH) (Yan and Mao, 2021). Moreover, pyruvate can be converted into acetaldehyde (HAc) through another pathway under the catalysis of pyruvate decarboxylase (PDC) and alcohol dehydrogenase (ADH), ultimately to ethanol (Chen et al., 2018). In the present study, *PDH* in aged quinoa seeds of L1 cultivar was upregulated, suggesting that the conversion of pyruvate to acetyl-CoA in L1 seeds might be promoted, thus providing more sufficient and stable substrates for TCA cycle. In addition, HAc was finally converted into ethanol through a series of reactions, owing to the upregulation of *ADH* and *acetate/butyrate-CoA ligase* (*AAE*), so as to achieve biological detoxification (**Figure 6B**).

TCA cycle is the key metabolic pathway for most organisms to harvest energy (Noctor et al., 2007). As one of the iconic pathways in plant metabolism, it is generally believed to not only be responsible for the oxidation of respiratory substrates to drive ATP synthesis, but also provide carbon skeletons for the anabolic processes and contribute to carbon-nitrogen interaction and stress responses (Zhang and Fernie, 2023). Many enzymes are involved in this process, such as aconitate hydratase (AH), isocitrate dehydrogenase (IDH), and succinate dehydrogenase (SDH), which strictly regulate the interconversion of organic acids in TCA cycle. AH catalyzes the reversible isomerization of citrate to produce isocitrate via cis-aconitate (Secgin et al., 2022), while IDH further catalyzes the oxidative decarboxylation of isocitrate to form 2-oxoglutarate and NADPH, which are closely related to ammonia assimilation and reactive oxygen species metabolism (Lemaitre et al., 2007). SDH plays a central role in mitochondrion to catalyze the conversion of succinate to fumarate, thereby connecting the TCA cycle and electron transport chain (Mao et al., 2018). In this study, *AH*, *IDH*, and *SDH* were partially induced by artificial aging in L1 quinoa seeds, suggesting that the TCA cycle might be activated to accelerate material metabolism and energy supply, thus providing guarantees for the subsequence germination of aged seeds (**Figures 2**, **6B**). Phosphoenolpyruvate carboxylase (PEPC), locating at the core of plant carbon metabolism, catalyzes the irreversible carboxylation of PEP to oxaloacetate (OAA) for TCA cycle (Zhao et al., 2018). Ectopic overexpression of *PEPC* in *Vicia narbonensis* improved nutrient status on seed maturation and altered channels carbon into organic acids, leading to greater seed storage capacity and increased protein content (Radchuk et al., 2007). In the present study, *PEPC* was upregulated in L1 aged quinoa seeds, manifesting that it might be beneficial for promoting the regulation of energy balance (**Figure 6B**). Glyoxylate, a small and very reactive dicarboxylic acid, is considered as a toxic intermediate (Lassalle et al., 2016). It can be reduced into glycolate under the catalysis of glyoxylate/hydroxypyruvate reductase (HPR), a dual activity enzyme. Furthermore, glycolate is either excreted or converted into hydroxy pyruvate (Mariyam et al., 2023). Herein, artificial aging might to some extent result in the accumulation and toxicity of glyoxylate in quinoa seeds, while *HPR* upregulation in L1 seeds was perhaps beneficial for clearing its toxicity, thereby maintaining the strong storage tolerance of L1 seeds (**Figure 6B**).

Additionally, it was reported in legumes that the bZIP transcription factor encoded by *ABSCISIC ACID INSENSITIVE 5* regulated the seed maturation and seed vigor (Zinsmeister et al., 2016). In *Arabidopsis thaliana*, the *abscisic acid insensitive 3* (*abi3*) and *abscisic acid deficient 1* mutants showed severely reduced traits of seed longevity and vigor, by affecting the downstream component of ABA signaling (Clerkx et al., 2004; Ooms et al., 1993). Recent years, a multiomic study of two rice cultivars with distinct seed vigor uncovered several transcription factors, such as bZIP23 and bZIP42, acted as the nodes of gene network in explaining vigor differences; overexpression of bZIP23 enhanced seed vigor, while its gene knockout reduced seed vigor (Wang et al., 2022). In this study, several bZIPs were identified in quinoa seeds under artificial accelerated aging (**Table 1**), which further indicated that bZIP transcription factors might play important roles in regulating seed vigor.

## 5 Conclusions

**Figure 7:**
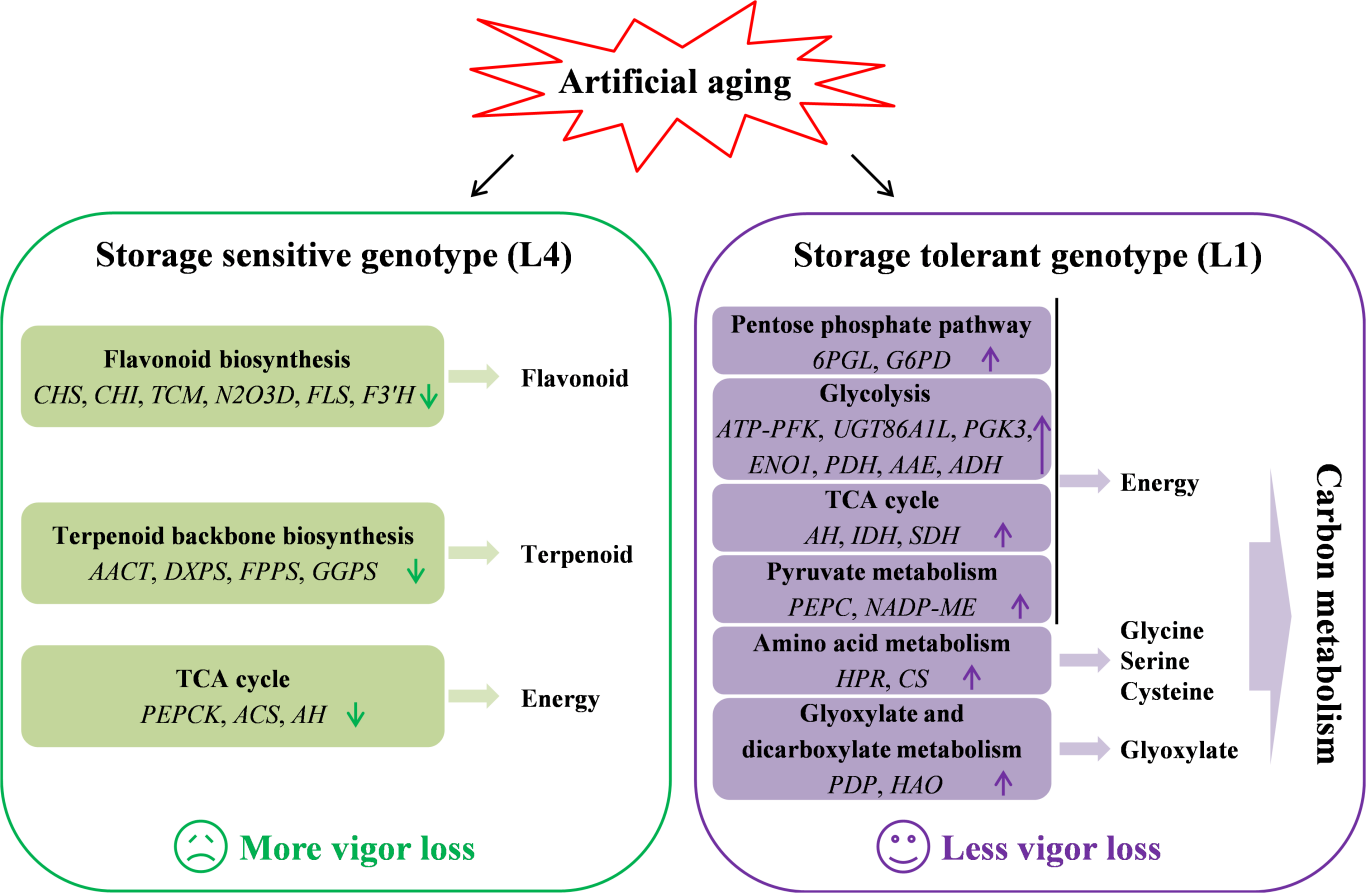
A putative model for seed vigor responsive mechanisms in quinoa under artificial accelerated aging.

In summary, multispectral imaging and germination phenotype showed that the effects of artificial aging on storage sensitive and tolerant seeds were obviously different, which manifested as L1 seeds having higher nCDA values and better germination performance than L4 seeds. Further combining multispectral imaging, germination phenotype, and transcriptomic sequencing, these two cultivars of quinoa seeds with different storage performance and vigor level were analyzed, and the possible mechanisms of seed vigor response were preliminarily uncovered (**Figure 7**). Transcriptomic analysis revealed the differences in metabolic pathways, especially, flavonoid biosynthesis, TCA cycle, and terpenoid backbone biosynthesis were significantly enriched in L4 seeds, while carbon metabolism pathway was significantly enriched in L1 seeds. It was also found that the expression levels of key candidate genes such as *CHS*, *CHI*, *AACT*, *ENO1*, *IDH*, *NADP-ME*, and *HAO2L* were significantly affected by artificial aging. Overall, the findings indicated that the alterations of flavonoids, terpenoids, carbon metabolism, and energy supply induced by aging were closely related to seed vigor loss in quinoa. This study elucidated the potential mechanisms of quinoa seeds vigor responses to aging at phenotypic and metabolic levels, thus providing a theoretical basis and possible scheme for the conservation of germplasm resources and the improvement of seed vigor in agricultural production.

## Data Availability

The RNA-seq raw data presented in the study are deposited in the NCBI SRA BioProject repository, accession number PRJNA1124963.

## Supplementary material

Supplementary Figure S1 Pearson correlation analysis between samples of L4 and L1 seeds after artificial accelerated aging.

Supplementary Figure S2 Top 20 significantly enriched GO terms of DEGs in pairwise comparison of L1/L4 under artificial accelerated aging. **(A)** Unaged control (L1A0/L4A0). **(B)** Aging for 1 d (L1A1/L4A1). **(C)** Aging for 2 d (L1A2/L4A2). **(D)** Aging for 4 d (L1A4/L4A4). BP, CC and MF represent biological process, cellular component and molecular function, respectively.

Supplementary Figure S3 The qRT-PCR validation of selected DEGs.

SUPPLEMENTARY TABLE S1 Primers for genes used in qRT-PCR analysis.

SUPPLEMENTARY TABLE S2 Data of morphological characteristics in L4 and L1 seeds after artificial accelerated aging treatments.

SUPPLEMENTARY TABLE S3 Summary of transcriptome data.

SUPPLEMENTARY TABLE S4 The expression and annotation of DEGs during artificial accelerated aging.

SUPPLEMENTARY TABLE S5 The information of GO analysis of DEGs after artificial accelerated aging treatment.

SUPPLEMENTARY TABLE S6 The information of KEGG analysis of DEGs after artificial accelerated aging treatment.

SUPPLEMENTARY TABLE S7 The significantly enriched KEGG pathways identified through the pairwise comparisons of artificial accelerated aging time.

SUPPLEMENTARY TABLE S8 The expression and annotation of specific and common TFs during artificial accelerated aging.
