# Supplementary figures and images for: Integrated multispectral imaging, germination phenotype, and transcriptomic analysis provide insights into seed vigor responsive mechanisms in quinoa under artificial accelerated aging
Source: Front Plant Sci. 2024 Sep 30;15:1435154. doi: 10.3389/fpls.2024.1435154 (PMC11471491; doi:10.3389/fpls.2024.1435154)

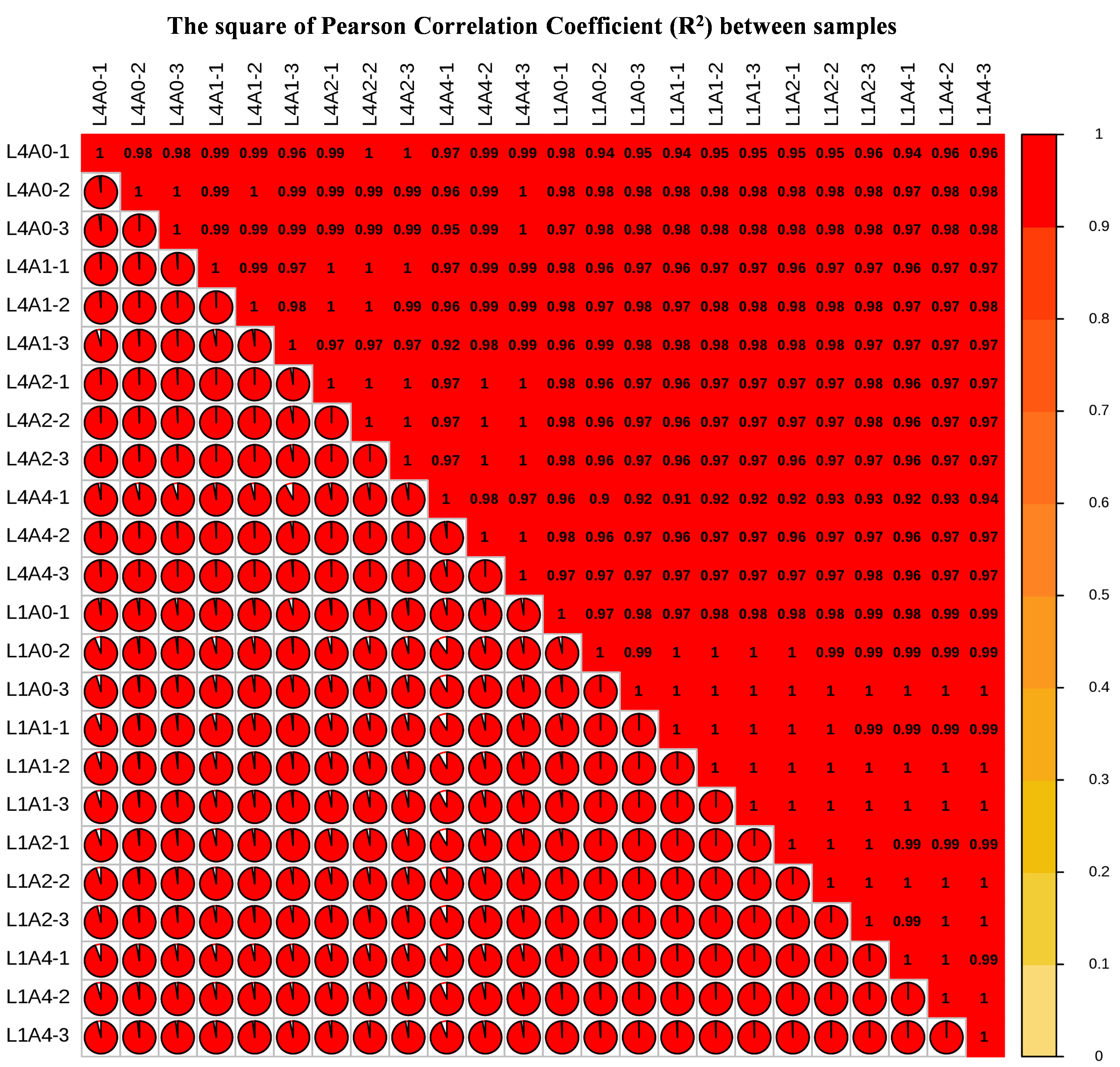

Supplement: Supplementary Figure S1 — Pearson correlation analysis between samples of L4 and L1 seeds after artificial accelerated aging. [file DataSheet1.zip › Supplementary Figure S1.TIF]

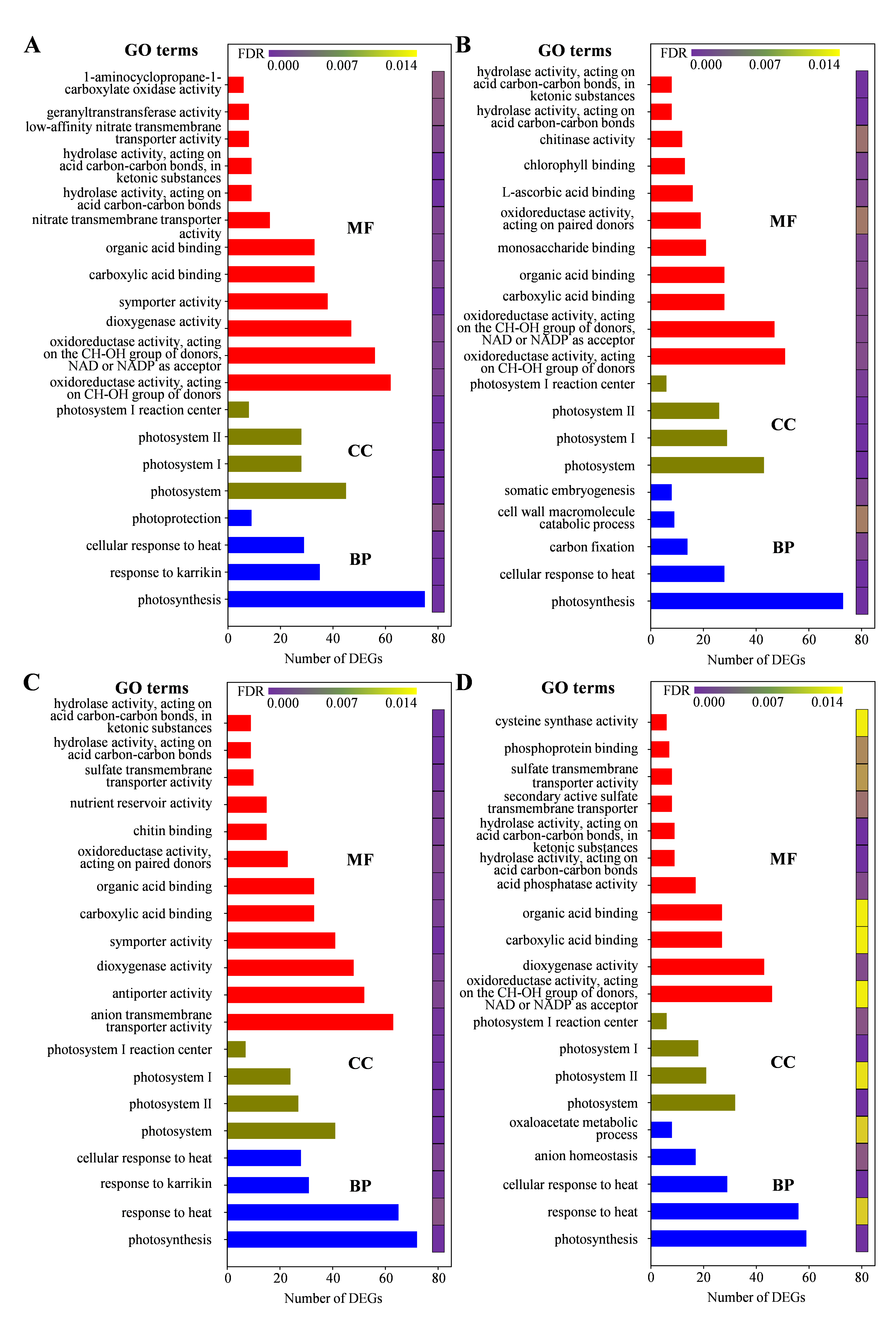

Supplement: Supplementary Figure S1 — Pearson correlation analysis between samples of L4 and L1 seeds after artificial accelerated aging. [file DataSheet1.zip › Supplementary Figure S2.TIF]

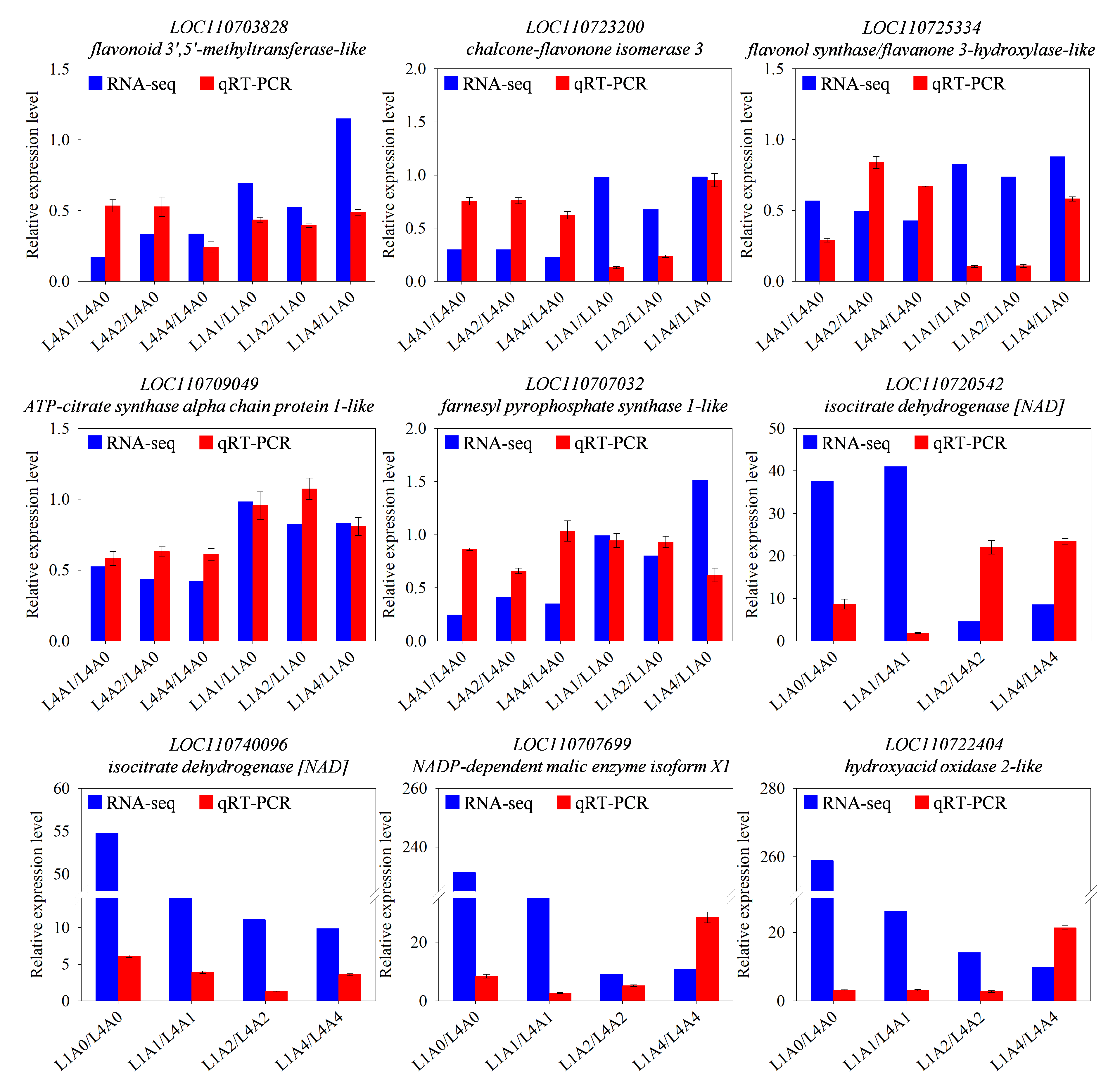

Supplement: Supplementary Figure S1 — Pearson correlation analysis between samples of L4 and L1 seeds after artificial accelerated aging. [file DataSheet1.zip › Supplementary Figure S3.TIF]
